# Associations Between Adult Triceps Skinfold Thickness and All-Cause, Cardiovascular and Cerebrovascular Mortality in NHANES 1999–2010: A Retrospective National Study

**DOI:** 10.3389/fcvm.2022.858994

**Published:** 2022-05-10

**Authors:** Weiya Li, Han Yin, Yilin Chen, Quanjun Liu, Yu Wang, Di Qiu, Huan Ma, Qingshan Geng

**Affiliations:** ^1^Department of Cardiology, Guangdong Cardiovascular Institute, Guangdong Provincial People's Hospital, Guangdong Academy of Medical Sciences, Guangzhou, China; ^2^School of Medicine, South China University of Technology, Guangzhou, China; ^3^Department of Anesthesiology, The First Affiliated Hospital of Zhengzhou University, Zhengzhou, China

**Keywords:** triceps skinfold, NHANES, all-cause mortality, cardiovascular mortality, cerebrovascular disease

## Abstract

**Background:**

The association between triceps skinfold (TSF) thickness and mortality in previous studies was controversial. This study aimed to explore how TSF thickness affects all-cause, cardiovascular, and cerebrovascular mortality among the United States (U.S.) general population.

**Methods:**

Our research included 25,954 adults in the National Health and Nutrition Examination Survey (NHANES) from 1999 to 2010. Participants were categorized by the baseline TSF quartiles [quartile 1 (Q1): < 11.8, (Q2): 11.8–17.4, (Q3): 17.4–25, and (Q4): ≥25; unit: millimeter (mm)]. Cox regression models were used to assess the association of TSF with all-cause, cardiovascular, and cerebrovascular mortality. The association between mid-arm muscle circumference (MAMC) and mortality was also explored. Subgroup analyses were conducted to assess heterogeneity in different subgroups.

**Results:**

The highest TSF group (Q4) had the lowest risk to experience all-cause (HR, 0.46; 95% CI, 0.38–0.59; *P* < 0.001) and cardiovascular mortality (HR, 0.35; 95% CI, 0.23–0.54; *P* < 0.001) than the lowest TSF group (Q1) after multivariate adjustment. However, there was no relationship between TSF quartiles and cerebrovascular mortality (HR, 0.98; 95%CI, 0.42–2.30; *P* = 0.97). The protective effects of TSF thickness on mortality still existed after adjusting for BMI and MAMC. For every 1 mm increase in TSF thickness, the risk of all-cause and cardiovascular death decreased by 4% (HR, 0.96; 95% CI, 0.95–0.97; *P* < 0.001) and 6% (HR, 0.94; 95% CI, 0.93–0.96; *P* < 0.001), respectively. In the stratified analysis, the relationships between TSF and mortality risk were generally similar across all subgroups.

**Conclusions:**

Higher TSF thickness was associated with lower all-cause and cardiovascular mortality, independent of BMI and MAMC. Our study revealed that the TSF thickness may be a convenient and credible indicator to predict mortality, especially in those with severe cardiovascular diseases.

## Introduction

Overweight and obesity are generally considered to be vital risk factors for cardiovascular and cerebrovascular disease (1–4), multiple cancers (5, 6), and various reasons for death (7, 8). Many studies have manifested body mass index (BMI) may not credible enough to distinguish body muscle and fat content (9–12). Variations in fat distribution (subcutaneous and visceral) may contribute to significant disease risk heterogeneity at any given BMI level, raising concerns about the ability of BMI to predict mortality (8, 13, 14).

Skinfold thickness has the advantage of representing the distribution of fat (15–17). Relationships between mortality and anthropometric indicators such as subscapular skinfold thickness, thigh, waist, and arm circumference have been reported (17–19). Triceps skinfold (TSF) thickness is an economical and convenient measurement to assess trunk and overall obesity. TSF could be used as a reasonable surrogate to investigate the relationship between subcutaneous fat and mortality (20). However, the relationship between TSF thickness and all-cause mortality is debatable in the existing studies (13, 20–22). Besides, there are very few studies to explore its associations with cardiovascular and cerebrovascular mortality.

This study is designed to explore the relationship of TSF thickness with all-cause, cardiovascular, and cerebrovascular mortalities in U.S. adults through a national survey—the National Health and Nutrition Examination Survey (NHANES) from 1999 to 2010. At the same time, mid-arm muscle circumference (MAMC) and mid-arm measurements (MUAC), which could reflect the muscle mass, were also included in our analysis as the secondary research objectives.

## Methods

### Study Design and Participants

The NHANES of the Centers for Disease Control (CDC) is a cross-sectional survey aimed to collect data about the health, nutritional status, and health behaviors of the noninstitutionalized civilian resident population in the U.S. (23). The NHANES data are released to the public in 2-year cycles. A multistage probability sampling design is used to ensure its representation of all of the United States civilian population. The protocol of NHANES was in line with the Health and Human Services (HHS) Policy for Protection of Human Research Subjects and approved by the National Center for Health Statistics (NCHS). All participants had signed a written informed consent before being incorporated into the NHANES. We excluded participants who were pregnant, breast-feeding, and suffering from any cancer at baseline. Those with ages less than 18 years old, missing anthropometric measurements data and mortality data were also removed from our research. Finally, a total of 25,954 participants from NHANES (1999–2010) were included in our study.

### Anthropometric Measurements Data

The anthropometry component data were precisely measured and recorded by well-trained NHANES health technologists and recorders based on standardized examination protocols. All these were done to ensure the data differences could reflect true differences in the NHANES body measurement values rather than technician and protocol variability or measurement error.

Holtain skinfold caliper with an accurate measurement up to a maximum of 45.0 mm was used to measure TSFs. Measurement steps are as follows. The NHANES staff grasped a fold of skin and subcutaneous adipose tissue approximately 2.0 cm above the mid-arm circumference mark. Place the tips of the caliper jaws over the complete skinfold and then release the caliper handle to exert full tension on the skinfold. Read the thickness of the closest to 0.1 mm. Each TSF consists of a double thickness of skin and underlying adipose tissue. The measurement will not continue if a fold that has two thicknesses of skin and underlying fat cannot be constructed.

Wrapping the measuring tape around the arm at the level of the upper arm mid-point mark to get the measured data of MUAC. Drawing a marker on the uppermost lateral border of the right ilium, the waist circumference was measured with a tape horizontally around the marker. All the measurements were taken to the nearest 0.1 centimeters (cm). We also got the MAMC by a standard calculation: (24) MAMC (cm) = MUAC (cm) -π× (TSF thickness [millimeters] ÷ 10).

### Other Data Collection

The physical and laboratory examinations were executed by professional test personnel to acquire the information on weight, height, total cholesterol (TC), high-density lipoprotein cholesterol (HDL-C), low-density lipoprotein cholesterol (LDL-C), and serum creatinine. We obtained BMI *via* weight (kilograms) divided by height (meters) squared. The estimated glomerular filtration rate (eGFR) was counted according to the Modification of Diet in Renal Disease (MDRD) formula (25). The race was described as white and non-white. Participants who had been diagnosed with diabetes and currently under used insulin or oral hypoglycemic drugs were considered to be combined with diabetes. Medical history of cardiovascular disease was defined as experiencing anyone of the four following events: coronary heart disease, heart failure, and angina pectoris. The stroke history was obtained by questioning participants “Has a doctor or other health professional ever told you that you had a stroke.”

### Follow Up and Mortality Data

The average follow-up time was about 119 months. The condition of death in 1999–2010 NHANES was extracted from the mortality file which involved a probabilistic match between NHANES and National Death Index (NDI) records prepared by the NCHS. Deaths from any cause were included in all-cause mortality. Cardiovascular (I00-I09, I11, I13, I20-I51) and cerebrovascular (I60–I69) mortality were defined according to the International Classification of Diseases, 10th Clinical Modification (ICD-10) System codes. Individuals were regarded as being alive if they did not have any match with death records in the follow-up period. All information about death and other variables can be accessed by visiting this web page (https://www.cdc.gov/nchs/nhanes/index.htm).

### Statistical Analyses

#### Baseline Description

Participants were divided into four groups according to the quartiles of TSF thickness. Continuous and categorical baseline statistics were described as means (SD), median (interquartile range), or number and percentage when appropriate. According to the types of data, one-way ANOVA, chi-square tests, Kruskal–Wallis *H*-test, or Fisher's test were utilized to analyze the differences among TSF quartiles.

#### Cox Risk Model Analysis

We used Cox proportional hazards models to evaluate the relationships between TSF thickness quartiles and all-cause, cardiovascular, and cerebrovascular mortality. Extra analyses of the impact of MAMC and MUAC on three kinds of mortality were also performed. The lowest quartiles were regarded as the reference and the three mid-arm measurements were treated as continuous variables when assessing the effect estimates of decreasing mortality for per millimeter (mm) or cm increment. In total, four sorts of Cox regression models were established. Model 1 was adjusted with age and gender. Model 2 was adjusted for multivariate variables, namely, age, gender, race, waist circumference, education level, marital status, smoking, HDL-C, TC, eGFR, and comorbidities (hypertension, diabetes, stroke, and cardiovascular disease). Model 3 included all variables in model 2 and BMI. Model 4 included MAMC and model 3.

#### Subgroup Analyses

We extra executed subgroup analyses based on gender (male or female), age (<65 or ≥65 years), race (white or non-white), and BMI (18.5–25 or <18.5, ≥25 kg/m^2^) to explore potential heterogeneity. First, we adjusted all covariables in model 3 except the subgroup variable itself. Then, we additionally adjusted MAMC in the subgroup analysis. *P* for interaction was counted with multiplicative terms by multiplying TSF by corresponding classified variables. In any subgroup Cox regression analysis, we considered TSF thickness as a continuous variable.

All data analyses were executed with SPSS version 25, and the level of significance was set at *P* < 0.05.

## Results

### Baseline Characteristics

**Figure 1:**
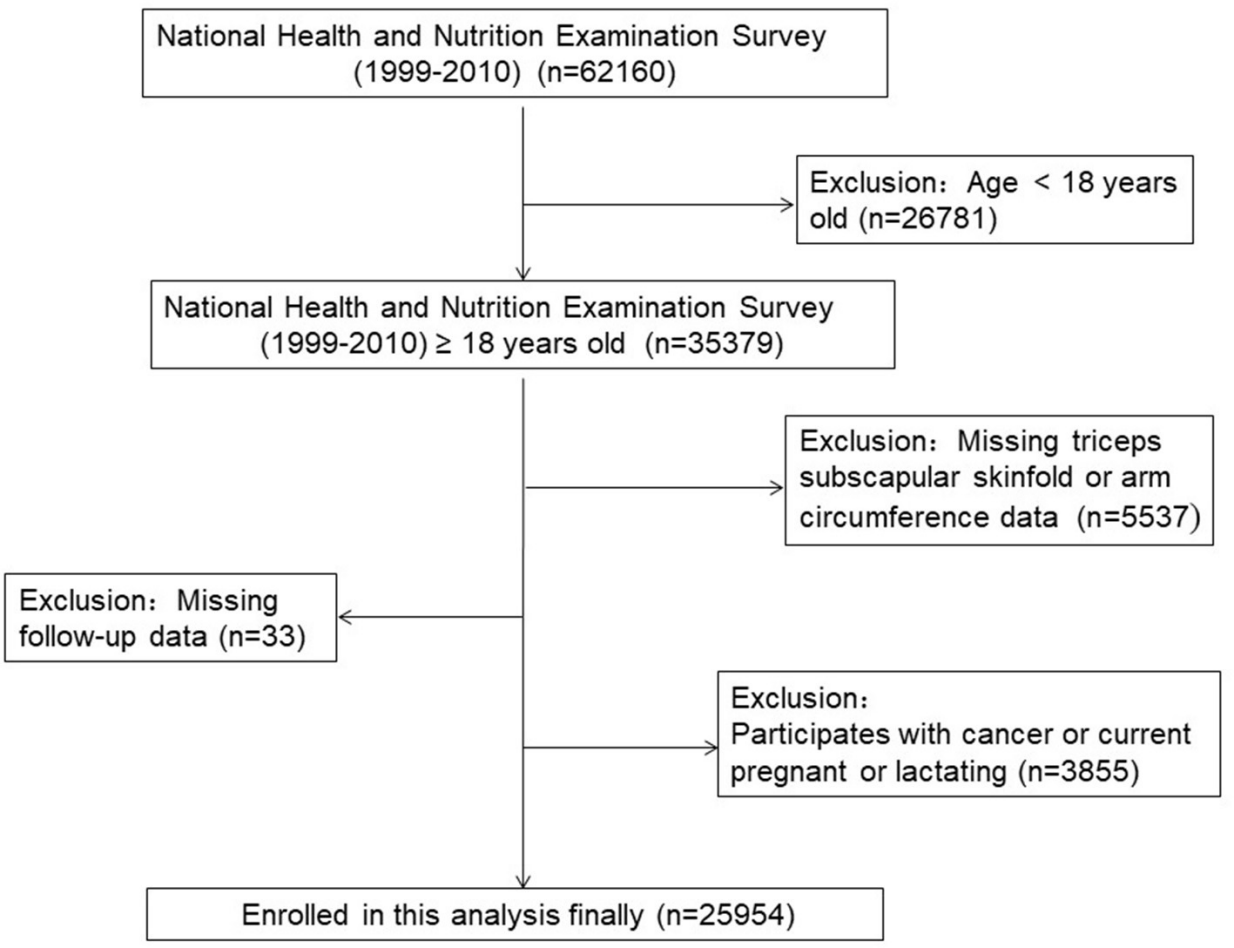
The research flow chart.

A total of 62,160 participants in NHANES from 1999 to 2010 were enrolled. The survival status of participants was tracked till 31 December 2015. Of these, 25,954 adult participants (52.8% males) from NHANES were included in our final analyses. The exclusion criteria were applied in Figure 1 and Supplementary Table 1. And the average age of all participants was 46.1±19.3 years. In total, 11,771 (45.4%) participants were white people. There were 9.3%, 9.9%, 2.9%, and 7.3% of all participants with a medical history of diabetes, hypertension, stroke, and cardiovascular diseases, respectively. After a follow-up of 119.5 ± 45.3 months, 3,507 (13.5%) participants experienced all-cause death, 629 (2.4%) participants experienced cardiovascular death, and 154 (0.6%) participants dead from cerebrovascular disease. In the study population, the average TSF thickness was 18.7 ± 8.5 mm. Women have much higher TSF thickness than men (23.6 ± 7.5 mm vs 14.3 ± 6.8 mm). The mean BMI, MAMC, and MUAC were 27.6 ± 3.4 kg/m^2^, 18.7 ± 8.5 mm, 26.4 ± 4.1 cm, and 32.3 ± 4.6 cm, respectively. Table 1 showed the baseline characteristics across the quartiles of TSF thickness.

**Table 1:** Baseline characteristics of different TSF thickness groups among study population.

**Characteristic**	**Overall**	**TSF quartiles, mm**				***P*** **-value**
		**Q1 ≤11.8**	**11.8 < Q2 ≤17.4**	**17.4 < Q3 ≤25**	**25 < Q4**	
**Number**	**25,954**	**6,645**	**6,377**	**6,619**	**6,313**	
**Demographic**						
Age, year	46.1 ± 19.3	43.6 ± 19.9	46.7 ± 19.9	47.3 ± 19.5	46.7 ± 17.6	< 0.001
Age ≥ 65, *n* (%)	5,420 (20.9)	1,246 (18.8)	1,481 (23.2)	1,552 (23.4)	1,141 (18.1)	< 0.001
Male, *n* (%)	13,706 (52.8)	5,929 (89.2)	4,228 (66.3)	2,440 (36.9)	1,109 (17.6)	< 0.001
Race, *n* (%)						< 0.001
White	11,771 (45.4)	2,839 (42.7)	3,030 (47.5)	3,155 (47.7)	2,747 (43.5)	
Non-white	14,183 (54.6)	3,806 (57.3)	3,347 (52.5)	3,464 (52.3)	3,566 (56.5)	
MAMC, cm	26.4 ± 4.1	27.4 ± 3.7	26.9 ± 4.2	25.6 ± 4.2	25.7 ± 4.1	< 0.001
MUAC, cm	32.3 ± 4.6	26.7 ± 1.9	30.7 ± 0.8	33.9 ± 0.9	38.3 ± 2.8	< 0.001
Waist circumference, cm	95.3 ± 15.0	86.9 ± 11.6	93.7 ± 13.4	96.9 ± 14.5	104.1 ± 15.0	< 0.001
BMI, kg/m^2^	27.6 ± 5.7	23.8 ± 3.7	26.3 ± 4.3	28.1 ± 4.9	32.4 ± 6.0	< 0.001
Smoked ≥ 100 cigarettes, *n* (%)	11,357 (43.8)	3,320 (50.0)	2,979 (46.7)	2,692 (40.7)	2,366 (37.5)	< 0.001
Less than high school graduated, *n* (%)	8,346 (32.3)	2,416 (36.4)	1,989 (31.2)	2,029 (30.7)	1,912 (30.3)	< 0.001
Marital status-married, *n* (%)	12,457 (48.0)	2,925 (44.0)	3,230 (50.7)	3,204 (48.4)	3,098 (49.1)	< 0.001
**Medical history**						
Diabetes, *n* (%)	2,407 (9.3)	399 (6.0)	537 (8.4)	698 (10.5)	773 (12.2)	< 0.001
Hypertension, *n* (%)	2,560 (9.9)	578 (8.7)	652 (10.2)	694 (10.5)	636 (10.1)	0.003
Stroke, *n* (%)	752 (2.9)	155 (2.3)	182 (2.9)	212 (3.2)	203 (3.2)	0.007
Cardiovascular diseases, *n* (%)	1,890 (7.3)	435 (6.5)	530 (8.3)	463 (7.0)	462 (7.3)	0.001
**Laboratory test**	
TC, mg/dl	196.3 ± 42.6	189.7 ± 44.0	196.6 ± 41.6	198.5 ± 42.8	200.5 ± 41.0	< 0.001
LDL-C, mg/dl	116.1 ± 36.0	112.4 ± 37.8	116.1 ± 34.9	116.8 ± 35.8	119.0 ± 35.3	< 0.001
HDL-C, mg/dl	52.3 ± 15.7	52.9 ± 15.9	51.3 ± 15.8	52.8 ± 16.0	52.1 ± 15.0	< 0.001
eGFR, mg/min/1.73 m^2^	97.5 ± 30.4	97.8 ± 27.8	96.8 ± 30.1	97.8 ± 31.9	97.6 ± 31.6	0.022
**Outcomes, *n* (%)**						
Cardiovascular disease mortality	629 (2.4)	209 (3.1)	178 (2.8)	143 (2.2)	99 (1.6)	< 0.001
Cerebrovascular disease mortality	154 (0.6)	41 (0.6)	32 (0.5)	46 (0.7)	35 (0.6)	0.514
All-cause mortality	3,507 (13.5)	1,038 (15.6)	975 (15.3)	882 (13.3)	612 (9.7)	< 0.001

*TSF, triceps skinfold; Q, quartiles; MAMC, mid-arm muscle circumference; MUAC, mid-upper arm circumference; BMI, body mass index; TC, total cholesterol; HDL-C, high-density lipoprotein cholesterol; LDL-C, low-density lipoprotein cholesterol; eGFR, estimated glomerular filtration rate*.

### The Relationship Between TSF Thickness and All-Cause, Cardiovascular, and Cerebrovascular Diseases Mortality

**Figure 2:**
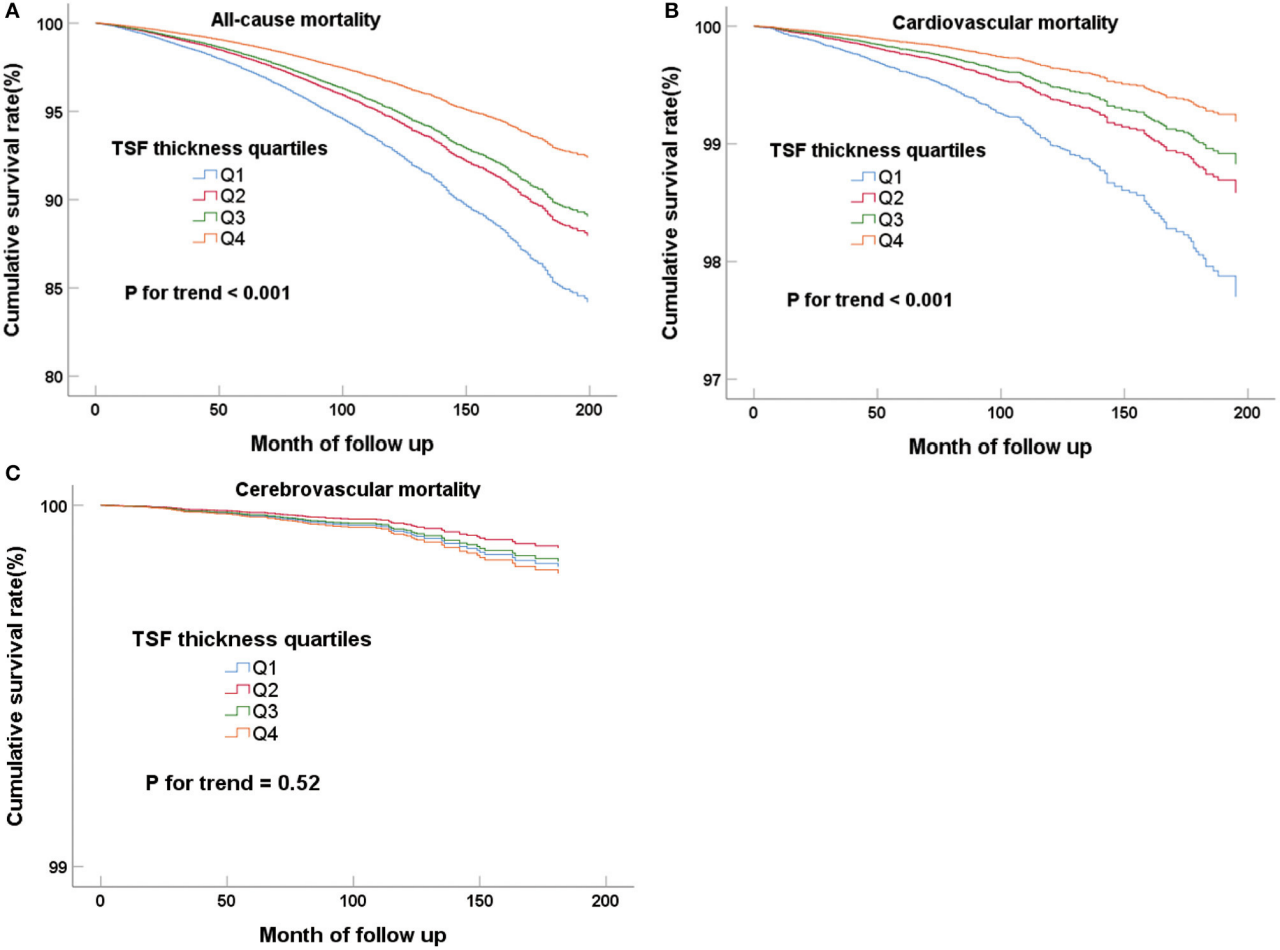
Cox regression curves for all-cause **(A)**, cardiovascular **(B)**, and cerebrovascular **(C)** mortality according to the quartiles of adult TSF after adjusting for model 4.

When the mid-arm measurements (TSF thickness, MAMC, and MUAC) were regarded as classified variables, the higher quartiles had the lower all-cause and cardiovascular mortality in all Cox regression models, and all the trends of the classifications of quartiles were statistically significant (*P* for trend < 0.05) (Table 2 for TSF; Supplementary Table 2 for MAMC; and Supplementary Table 3 for MUAC). The Cox regression curves adjusted for multivariate variables (model 3) were shown in Figure 2 and Supplementary Figures 1, 2. The highest TSF group (Q4) is more likely to experienced all-cause (HR, 0.64; 95%CI, 0.54–0.76; *P* < 0.001) and cardiovascular mortality (HR, 0.54; 95%CI, 0.36–0.79; *P* = 0.002) than the lowest group (Q1) in model 3 after adjustment for gender, age, race, waist circumference, education level, marital status, smoking, HDL-C, TC, eGFR, comorbidities (hypertension, diabetes, stroke, and cardiovascular disease), and BMI. However, there was no relationship between TSF and cerebrovascular mortality in model 3 (HR, 0.98; 95%CI, 0.42–2.30; *P* = 0.97). After putting MAMC into model 3, we found the incorporation of TSF and MAMC significantly improved the performance of the Cox proportional hazards model (model 4) in predicting all-cause and cardiovascular mortality (*P* < 0.001).

**Table 2:** Cox regression analysis between TSF and all-cause, cardiovascular, and cerebrovascular mortality.

	**Unadjusted**	**Model 1**	**Model 2**	**Model 3**	**Model 4**
	**HR(95% CI),** ***P*** **value**	**HR(95% CI),** ***P*** **value**	**HR(95% CI),** ***P*** **value**	**HR(95% CI),** ***P*** **value**	**HR(95% CI),** ***P*** **value**
**All-cause mortality**					
TSF (per mm increment)	0.979 (0.975, 0.983) < 0.001	0.979 (0.974, 0.984) < 0.001	0.972 (0.965, 0.980) < 0.001	0.98 (0.97, 0.99) < 0.001	0.96 (0.95, 0.97) < 0.001
**Triceps skinfold quartiles**					
Q1	Reference	Reference	Reference	Reference	Reference
Q2	0.99 (0.91, 1.08) 0.84	0.84 (0.77, 0.91) < 0.001	0.80 (0.71, 0.89) < 0.001	0.81 (0.72, 0.90) < 0.001	0.75 (0.67, 0.84) < 0.001
Q3	0.86 (0.79, 0.94) 0.001	0.78 (0.71, 0.86) < 0.001	0.77 (0.67, 0.88) < 0.001	0.81 (0.71, 0.93) 0.002	0.68 (0.59, 0.78) < 0.001
Q4	0.62 (0.56, 0.68) < 0.001	0.65 (0.58, 0.73) < 0.001	0.58 (0.49, 0.68) < 0.001	0.64 (0.54, 0.76) < 0.001	0.46 (0.38, 0.59) < 0.001
*P* for trend	< 0.001	< 0.001	< 0.001	< 0.001	< 0.001
**Cardiovascular mortality**					
TSF (per mm increment)	0.97 (0.96, 0.98) < 0.001	0.98 (0.97, 0.995) 0.005	0.97 (0.95, 0.99) < 0.001	0.97 (0.95, 0.99) 0.001	0.94 (0.93, 0.96) < 0.001
**Triceps skinfold quartiles**					
Q1	Reference	Reference	Reference	Reference	Reference
Q2	0.90 (0.73, 1.10) 0.28	0.79 (0.65, 0.97) 0.023	0.67 (0.52, 0.86) 0.002	0.67 (0.52, 0.87) 0.002	0.62 (0.48, 0.79) < 0.001
Q3	0.69 (0.56, 0.86) 0.001	0.74 (0.59, 0.93) 0.01	0.63 (0.47, 0.85) 0.003	0.64 (0.47, 0.87) 0.004	0.51 (0.37, 0.70) < 0.001
Q4	0.49 (0.39, 0.63) < 0.001	0.70 (0.53, 0.92) 0.009	0.50 (0.34, 0.73) < 0.001	0.54 (0.36, 0.79) 0.002	0.35 (0.23, 0.54) < 0.001
*P* for trend	< 0.001	0.02	0.002	0.004	< 0.001
**Cerebrovascular mortality**					
TSF (per mm increment)	1.00 (0.98, 1.01) 0.65	1.00 (0.98, 1.13) 0.86	1.00 (0.96, 1.04) 0.86	0.99 (0.95, 1.03) 0.74	1.00 (0.95, 1.04) 0.91
**Triceps skinfold quartiles**					
Q1	Reference	Reference	Reference	Reference	Reference
Q2	0.82 (0.52, 1.31) 0.41	0.68 (0.42, 1.08) 0.10	0.65 (0.35, 1.20) 0.17	0.68 (0.37, 1.26) 0.22	0.70 (0.37, 1.30) 0.26
Q3	1.14 (0.75, 1.74) 0.54	1.05 (0.66, 1.67) 0.84	0.81 (0.41, 1.61) 0.55	0.85 (0.43, 1.71) 0.65	0.92 (0.44, 1.89) 0.81
Q4	0.89 (0.57, 1.40) 0.61	1.04 (0.62, 1.76) 0.88	1.02 (0.46, 2.28) 0.96	0.98 (0.42, 2.30) 0.97	1.11 (0.44, 2.79) 0.82
*P* for trend	0.5	0.23	0.41	0.55	0.52

*Model 1: adjusted for age and gender*.

*Model 2: adjusted for multivariate variables: age, gender, race, waist circumference, education level, marital status, smoking, HDL-C, TC, eGFR, and comorbidities (hypertension, diabetes, stroke, and cardiovascular disease)*.

*Model 3: adjusted for model 2 and BMI*.

*Model 4: adjusted for model 3 and MAMC*.

*Q, quartiles; HR, hazard ratio; CI, confidence interval*.

When the mid-arm measurements (TSF thickness, MAMC, and MUAC) were evaluated on a continuous scale, the negative correlations between them and all-cause and cardiovascular mortality still existed. First, the TSF thickness was negative correlated with all-cause (Table 2; HR, 0.96; 95%CI, 0.95–0.97; *P* < 0.001) and cardiovascular mortality (Table 2; HR, 0.94; 95%CI, 0.93–0.96; *P* < 0.001) after full adjustments, namely, gender, age, race, waist circumference, education level, marital status, smoking, HDL-C, TC, eGFR, comorbidities (hypertension, diabetes, stroke, and cardiovascular disease), BMI, and MAMC (model 4). This represented per mm increment of TSF thickness could decrease 4% and 6% risk of the all-cause and cardiovascular mortality, respectively. Similar results were observed in the analyses of MAMC and MUAC (Supplementary Tables 2, 3), in which one cm increase in MAMC and MUAC was associated with 10% and 11% risk reduction for all-cause mortality, and 13% and 14% risk reduction for cardiovascular mortality.

### Subgroup Analyses

**Figure 3:**
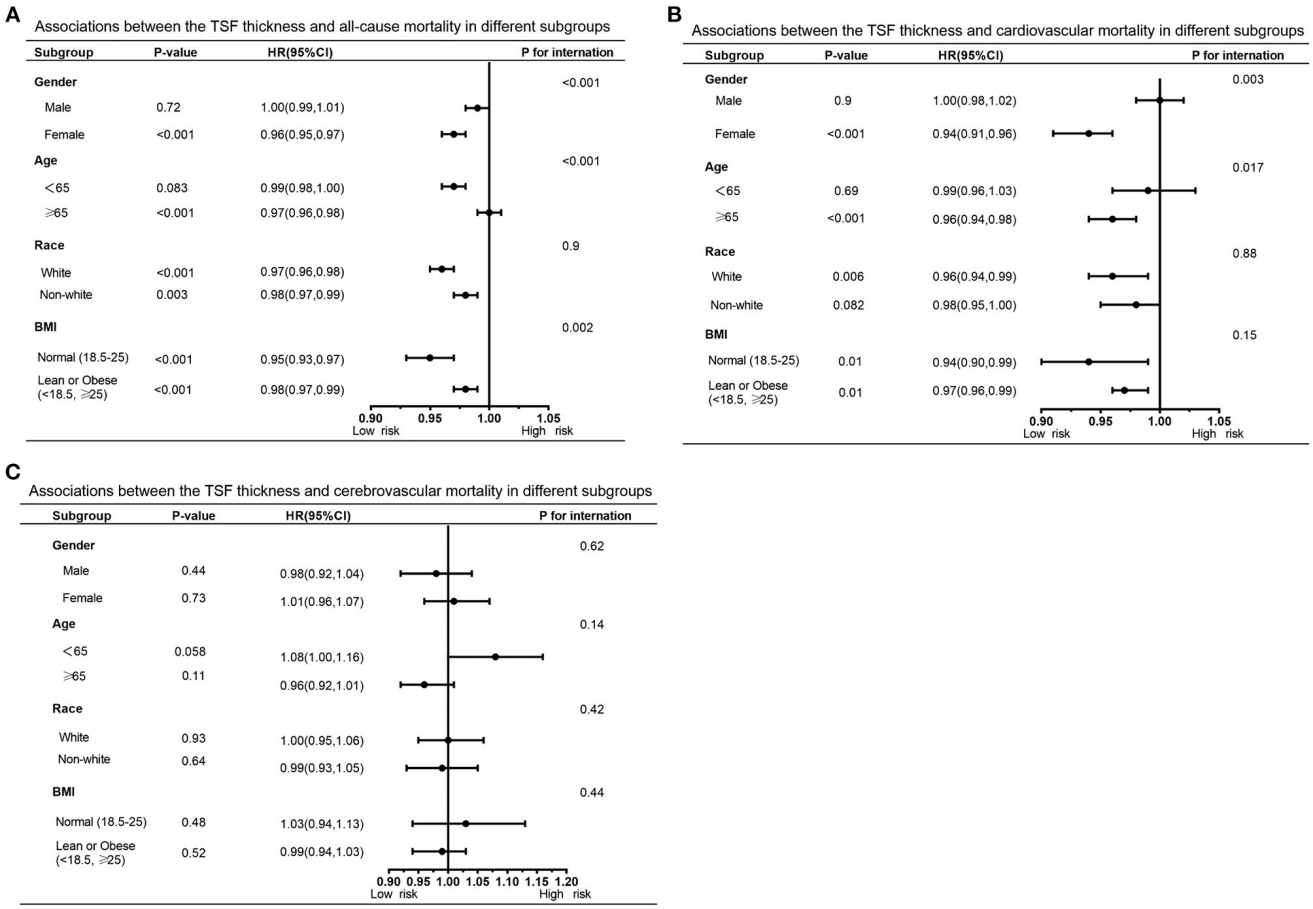
Forest graphs of associations of TSF with all-cause mortality **(A)** and cardiovascular mortality **(B)** stratified by gender, age, race, and BMI after adjusting for model 3.

**Figure 4:**
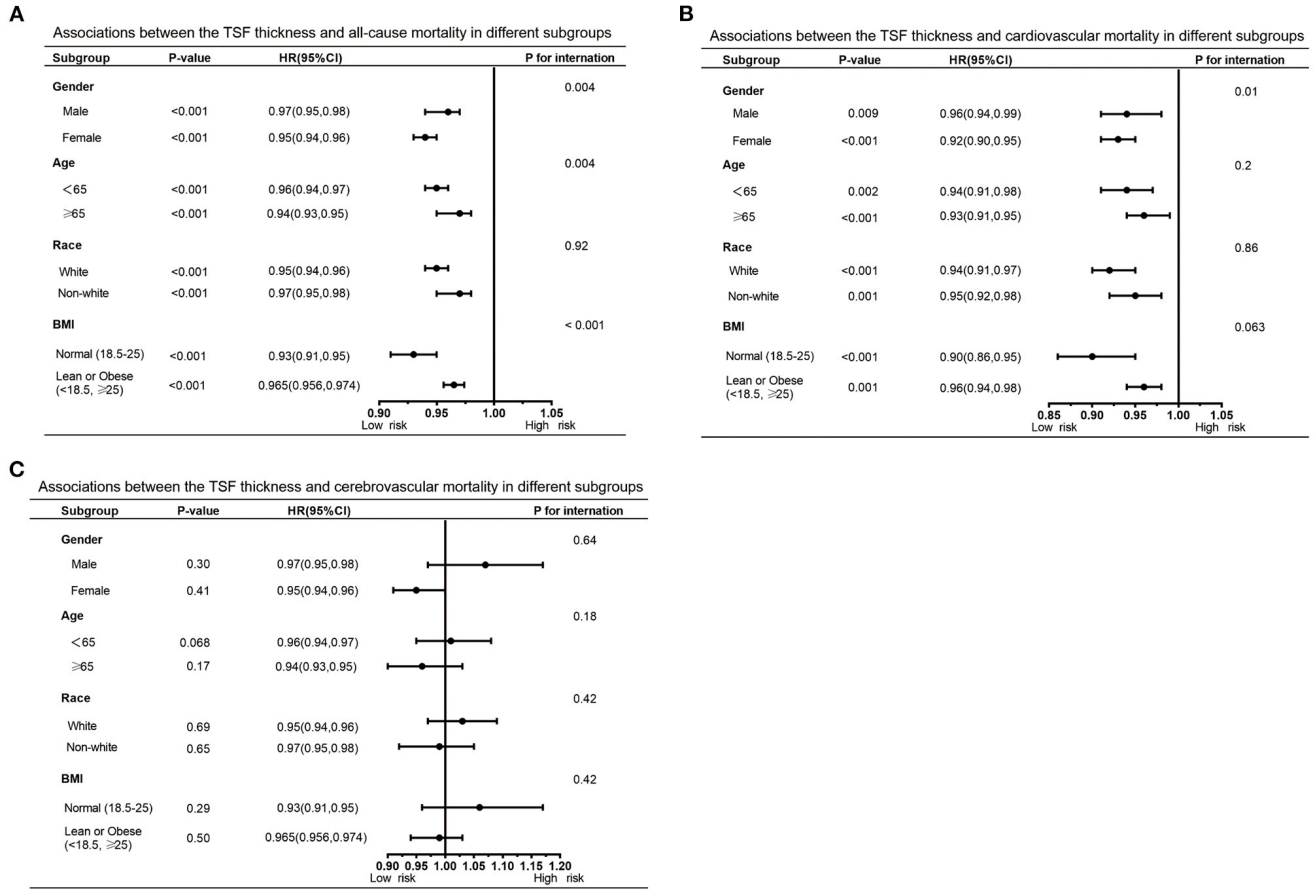
Forest graphs of associations of TSF with all-cause mortality **(A)** and cardiovascular mortality **(B)** stratified by gender, age, race, and BMI after adjusting for model 4.

Furthermore, we also performed subgroup analyses to explore potential heterogeneity between TSF thickness and three kinds of deaths, stratified by gender, age, race, and BMI (Tables 3, 4). In any subgroups, we considered TSF thickness as a continuous variable. The forest plots in Figures 3, 4 provided a simple and intuitive description of our subgroup analyses results. We found there were significant interactions between TSF thickness and gender (*P* < 0.001) and age (*P* < 0.001) for all-cause mortality and cardiovascular mortality before adjusting MAMC (Table 3; Figure 3). Both female participants and people whose age ≥ 65 years old with higher TSF thickness had lower risks of all-cause and cardiovascular mortality. Notably, after adjusting for MAMC, the relationships between TSF thickness and the three kinds of mortality risks were generally similar across subgroups by age (< 65 or ≥65 years), men and women, and race categories (white or non-white). In addition, thicker TSF was protective for all-cause and cardiovascular mortality in both normal (18.5 ≤ BMI < 25) and abnormal BMI (< 18.5, ≥25) groups, although the protective effect appeared stronger in the normal BMI group (Table 4; Figure 4).

**Table 3:** Subgroup analysis of TSF and all-cause, cardiovascular, and cerebrovascular mortality.

	**All-cause mortality**			**Cardiovascular mortality**			**Cerebrovascular mortality**		
	**No. of survival**	**HR (95% CI)**,	***P*** **for**	**No. of survival**	**HR (95% CI)**,	***P*** **for**	**No. of survival**	**HR (95% CI)**,	***P*** **for**
	**participants/deaths**	***P*** **value**	**interaction**	**participants/deaths**	***P*** **value**	**interaction**	**participants/deaths**	***P*** **value**	**interaction**
**Gender**			< 0.001			0.003			0.62
Male	11,659/2,047	1.00 (0.99, 1.01) 0.72		13,296/410	1.00 (0.98, 1.02) 0.90		13,625/81	0.98 (0.92, 1.04) 0.44	
Female	10,788/1,460	0.96 (0.95, 0.97) < 0.001		12,029/219	0.94 (0.91, 0.96) < 0.001		12,175/73	1.01 (0.96, 1.07) 0.73	
**Age, years**			< 0.001			0.017			0.14
< 65	19,335/1,199	0.99 (0.98, 1.00) 0.083		20,363/171	0.99 (0.96, 1.03) 0.69		20,499/35	1.08 (1.00, 1.16) 0.058	
≥65	3,112/2,308	0.97 (0.96, 0.98) < 0.001		4,962/458	0.96 (0.94, 0.98) < 0.001		5,301/119	0.96 (0.92, 1.01) 0.11	
**Race**			0.9			0.88			0.42
White	9,909/1,862	0.97 (0.96, 0.98) < 0.001		11,441/330	0.96 (0.94, 0.99) 0.006		11,685/86	1.00 (0.95, 1.06) 0.93	
Non-white	12,538/1,645	0.98 (0.97, 0.99) 0.003		13,884/299	0.98 (0.95, 1.00) 0.082		14,115/68	0.99 (0.93, 1.05) 0.64	
**BMI, kg/m** ^2^			0.002			0.15			0.44
Normal(18.5-25)	7,539/1,059	0.95 (0.93, 0.97) < 0.001		8,412/186	0.94 (0.90, 0.99) 0.01		8,555/43	1.03 (0.94, 1.13) 0.48	
Lean or Obese	14,844/2,336	0.98 (0.97, 0.99) < 0.001		16,761/419	0.97 (0.96, 0.99) 0.01		17,079/101	0.99 (0.94, 1.03) 0.52	
(< 18.5, ≥25)

*Adjusting for all variables in model 2 for subgroups analysis (excluding MAMC). When analyzing a subgroup variable, age, gender, race, waist circumference, education level, marital status, smoking, BMI, HDL-C, TC, eGFR, and comorbidities (hypertension, diabetes, stroke, and cardiovascular disease) were all adjusted except the variable itself*.

**Table 4:** Subgroup analysis of TSF and all-cause, cardiovascular, and cerebrovascular mortality.

	**All-Cause mortality**			**Cardiovascular mortality**			**Cerebrovascular mortality**		
	**No. of survival**	**HR (95% CI)**,	***P*** **for**	**No. of survival**	**HR (95% CI)**,	***P*** **for**	**No. of survival**	**HR (95% CI)**,	***P*** **for**
	**participants/deaths**	***P*** **value**	**interaction**	**participants/deaths**	***P*** **value**	**interaction**	**participants/deaths**	***P*** **value**	**interaction**
**Gender**			0.004			0.01			0.64
Male	11,659/2,047	0.97 (0.95, 0.98) < 0.001		13,296/410	0.96 (0.94, 0.99) 0.009		13,625/81	0.96 (0.90, 1.03) 0.30	
Female	10,788/1,460	0.95 (0.94, 0.96) < 0.001		12,029/219	0.92 (0.90, 0.95) < 0.001		12,175/73	1.03 (0.97, 1.09) 0.41	
**Age, years**			0.004			0.2			0.18
< 65	19,335/1,199	0.96 (0.94, 0.97) < 0.001		20,363/171	0.94 (0.91, 0.98) 0.002		20,499/35	1.07 (0.97, 1.17) 0.17	
≥65	3,112/2,308	0.94 (0.93, 0.95) < 0.001		4,962/458	0.93 (0.91, 0.95) < 0.001		5,301/119	0.95 (0.91, 1.00) 0.068	
**Race**			0.92			0.86			0.42
White	9,909/1,862	0.95 (0.94, 0.96) < 0.001		11,441/330	0.94 (0.91, 0.97) < 0.001		11,685/86	1.01 (0.95, 1.08) 0.69	
Non-white	12,538/1,645	0.97 (0.95, 0.98) < 0.001		13,884/299	0.95 (0.92, 0.98) 0.001		14,115/68	0.99 (0.92, 1.05) 0.65	
**BMI, kg/m** ^2^			< 0.001			0.063			0.42
Normal(18.5–25)	7,539/1,059	0.93 (0.91, 0.95) < 0.001		8,412/186	0.90 (0.86, 0.95) < 0.001		8,555/43	1.06 (0.96, 1.17) 0.29	
Lean or Obese	14,844/2,336	0.965 (0.956, 0.974) < 0.001		16,761/419	0.96 (0.94, 0.98) 0.001		17,079/101	0.99 (0.94, 1.03) 0.50	
(< 18.5, ≥25)

*When analyzing a subgroup variable, age, gender, race, waist circumference, MAMC, education level, marital status, smoking, BMI, HDL-C, TC, eGFR, and comorbidities (hypertension, diabetes, stroke, and cardiovascular disease) were all adjusted except the variable itself*.

## Discussion

In the large U.S. cohort data, we found that higher TSF thickness was associated with lower all-cause and cardiovascular mortality. Similar results were observed in the analyses of MAMC and MUAC. And the effects of risk reduction were stronger for cardiovascular mortality than for all-cause mortality. In all subgroups, the relationship between TSF thickness and mortality risks was generally similar. However, no significant correlation between the mid-arm measurements and cerebrovascular mortality was found.

Recent studies have proven that subcutaneous fat and fat to surrounding tissue and limbs are beneficial to the health of humans (14, 26). Adequate subcutaneous fat can indirectly regulate glucose and lipid metabolism and immune response by promoting the production of leptin to potentially benefit death reduction (27). More subcutaneous fat may prevent atherosclerosis and non-adipose tissue lipotoxicity by separating non-esterified fatty acids from food (28). Lack of inflation in subcutaneous adipose could lead to internal organs and ectopic fatty deposition, inflammatory, and insulin resistance (2, 3). Atherosclerotic cardiovascular disease (ASCVD) events have been significantly reduced because of the changes in diet structure and extensive use of established lipid-modifying drugs (29). Centrally distributed body fat, which can be reflected by skinfold thickness, is an important risk factor for coronary heart disease events (21, 30). Thus, a credible barometer that could reflect the skin adipose tissue of the mid-arm is warranted.

The “obesity paradox” and heterogeneity of disease risk have been widely reported when using BMI to assess adiposity (31–34). Epidemiological studies showed the association between obesity classified by standard BMI and all-cause mortality was a U-shaped curve in ordinary people, and the overweight populations were at the lowest point (35, 36). The difference in fat distribution may explain this risk heterogeneity we mentioned above. As a reliable index of mid-arm subcutaneous fat tissues, TSF thickness could well represent the distribution of peripheral fat (37). Therefore, TSF may be an important predictor of death in clinical practice.

However, disputes existed in the previous studies on TSF and adverse prognosis. Four studies revealed the positive relationships between TSF and all-cause mortality, ischemic heart disease, and stroke mortality (15, 21, 22, 38–40). Besides, other studies reported the opposite results in specific populations (13, 24, 41). Nevertheless, the existing studies have some limitations: such as small sample size, specific population (white males, hemodialysis patients, and older adults age ≥ 60 years old), and short follow-up time. Our study used the large U.S. cohort data to provide new evidence in this field.

To the best of our knowledge, this is the largest cohort study that proved higher TSF thickness is, independent of BMI, correlated with lower all-cause and cardiovascular mortality in the U.S. general population. Cardiovascular and cerebrovascular mortality are leading causes of death in the U.S. (42). Exploring the convenient and credible indicators to forecast mortality risk is vital for primary health care. Our results suggest that enhancing the TSF thickness may be a general approach to reducing mortality.

Mid-arm measurements (MUAC) are a reliable substitution of body mass or muscle mass (43). We found that MAMC enhance the capacity of the TSF model to predict mortality, which is consistent with the findings in a large Chinese cohort (20). MAMC significantly improved the performance of the Cox proportional hazards model in predicting all-cause and cardiovascular mortality.

Furthermore, before adjusting for MAMC, the TSF was associated with mortality only in the elderly and female subgroups, which is consistent with a previous study (41). The reason may be different causes of death. In the elderly, the deaths were more likely caused by chronic wasting and malignant diseases, while the deaths of young people were more likely caused by acute diseases, unintentional injuries, and suicide (42). As a surrogate marker for nutritional and health status, thicker TSF may protect against malnutrition and cachexia, thereby avoiding death from chronic and malignant diseases (44). Estrogen has been proven to impact survival by expanding subcutaneous fat (26). Besides, women always have a larger range of TSF thicknesses, resulting in better discrimination in statistics. All these reasons may lead to heterogeneity in age and gender subgroups. After adjusting for MAMC, the protective effects of TSF thickness were significantly increased in young and male subgroups. MAMC were found to be significantly positively associated with resting metabolic rate, which influences energy expenditure under pathological conditions (45). The resting metabolic rate is higher in young adults and men. Therefore, MAMC adjustment resulted in stronger protective effects in those participants.

There are several limitations to declare. First, the results of our study were based on the data from the U.S. population, and the findings need to be confirmed in other populations. Second, the change in TSF thickness was not considered during the follow-up. Third, self-reported medical conditions and smoking status of individuals may be affected by recall bias or misclassification. Fourth, despite a great quantity of potentially confounding factors having been adjusted, some undetected confounders still cannot be excluded. Based on the mode in which NHANES collected data, the pregnancy and lactation status were reported only among women in specific age groups, which resulted in missing data. Fifth, our study was a retrospective cohort study, so causal inferences cannot be made.

## Conclusion

In conclusion, in the large U.S. cohort data, we found that higher TSF thickness was associated with lower all-cause and cardiovascular mortality, independent of BMI and MAMC. Our study revealed that the TSF thickness may be a convenient and credible indicator to predict mortality. Further high-quality trials of early intervention of TSF thickness are required, especially in those with severe cardiovascular diseases.

## Data Availability Statement

The original contributions presented in the study are included in the article/Supplementary Material, further inquiries can be directed to the corresponding author/s.

## Ethics Statement

The studies involving human participants were reviewed and approved by National Center for Health Statistics. The patients/participants provided their written informed consent to participate in this study.

## Author Contributions

WL, DQ, HY, and QL: conceptualization and methodology. WL and YC: formal analysis. WL, HM, and QG: supervision and validation. WL, QG, and DQ: writing and revision. All authors contributed to the article and approved the submitted version.

## Funding

This study was supported by the grants of the National Key R&D Program of China (2018YFC2001805), the Natural Science Foundation of Guangdong Province (2019A1515011224), the Guangdong Medical Science and Technology Research Fund (2019118152336191), the Guangdong Provincial Bureau of Traditional Chinese Medicine (20201008), the High-level Hospital Construction Project of Guangdong Provincial People's Hospital (DFJH201811, DFJH201922, and DFJH2020003), and Leading Medical Talents in Guangdong Province (KJ012019431).

## Conflict of Interest

The authors declare that the research was conducted in the absence of any commercial or financial relationships that could be construed as a potential conflict of interest.

## Publisher's Note

All claims expressed in this article are solely those of the authors and do not necessarily represent those of their affiliated organizations, or those of the publisher, the editors and the reviewers. Any product that may be evaluated in this article, or claim that may be made by its manufacturer, is not guaranteed or endorsed by the publisher.
