# Supplementary material for: Associations Between Adult Triceps Skinfold Thickness and All-Cause, Cardiovascular and Cerebrovascular Mortality in NHANES 1999–2010: A Retrospective National Study
Source: Front Cardiovasc Med. 2022 May 10;9:858994. doi: 10.3389/fcvm.2022.858994 (PMC9127233; doi:10.3389/fcvm.2022.858994)
Supplement: Supplementary file 1 [file Data_Sheet_1.PDF]

Supplementary Material

1.1 Supplementary Tables

Supplementary Table 1 | The proportion of three kinds of deaths before and after removing special populations (cancer or pregnancy or lactation)

	Number of total people	Number of all-cause deaths (Death proportion)	Number of cardiovascular deaths (Death proportion)	Number of cerebrovascular deaths (Death proportion)
Overall	29824	4467 (15%)	769 (2.6%)	190 (1%)
Participates with cancer or pregnancy or lactation	3885	953 (24.7%)	138 (3.6%)	35 (0.9%)
Overall participates after deleting cancer, pregnancy and lactation	25954	3507 (13.5%)	629 (2.4%)	154 (0.6%)

Supplementary Table 2 | Cox regression analysis between MAMC and all-cause, cardiovascular as well as cerebrovascular mortality**

	Unadjusted<br>HR(95% CI),P-value	Model 1<br>HR(95% CI),P-value	Model 2<br>HR(95% CI),P-value	Model 3<br>HR(95% CI),P-value	Model 4<br>HR(95% CI),P-value
**All-cause mortality**					
MAMC (per cm increment)	0.97(0.96,0.98) <0.001	0.95(0.94,0.97) <0.001	0.93(0.92,0.95) <0.001	0.95(0.93,0.97) <0.001	0.90(0.88,0.92) <0.001
MAMC quartiles					
Q1	Reference	Reference	Reference	Reference	Reference
Q2	1.33(1.21,1.45) <0.001	0.92(0.84,1.01) 0.087	0.83(0.73,0.94) 0.003	0.87(0.77,0.99) 0.032	0.79(0.69,0.89) <0.001
Q3	1.10(1.00,1.20) 0.049	0.74(0.66,0.83) <0.001	0.67(0.58,0.77) <0.001	0.73(0.62,0.85) <0.001	0.60(0.51,0.70) <0.001
Q4	0.73(0.66,0.81) <0.001	0.66(0.59,0.75) <0.001	0.55(0.46,0.66) <0.001	0.64(0.53,0.78) <0.001	0.46(0.37,0.57) <0.001
P for trend	<0.001	<0.001	<0.001	<0.001	<0.001
**Cardiovascular mortality**					

MAMC (per cm increment)	0.98(0.96,0.998)	0.029	0.97(0.94,0.99)	0.014	0.92(0.89,0.96)	<0.001	0.93(0.89,0.97)	0.001	0.87(0.83,0.92)	<0.001
MAMC quartiles										
Q1	Reference		Reference		Reference		Reference		Reference	
Q2	1.47(1.18,1.82)	<0.001	0.89(0.70,1.12)	0.32	0.79(0.59,1.05)	0.11	0.81(0.60,1.08)	0.15	0.71(0.52,0.97)	0.024
Q3	1.20(0.96,1.50)	0.11	0.69(0.53,0.91)	0.007	0.59(0.42,0.83)	0.002	0.62(0.43,0.88)	0.007	0.48(0.33,0.69)	<0.001
Q4	0.86(0.67,1.09)	0.21	0.75(0.56,1.01)	0.057	0.52(0.34,0.79)	0.002	0.58(0.37,0.90)	0.015	0.37(0.23,0.61)	<0.001
P for trend	<0.001		0.036		0.007		0.038		0.001	
**Cerebrovascular mortality**										
MAMC (per cm increment)	0.95(0.91,0.99)	0.008	0.98(0.92,1.03)	0.40	1.00(0.92,1.10)	0.91	1.03(0.93,1.13)	0.61	1.02(0.92,1.04)	0.68
MAMC quartiles										
Q1	Reference		Reference		Reference		Reference		Reference	
Q2	1.39(0.93,2.08)	0.11	1.06(0.69,1.64)	0.79	1.08(0.59,1.99)	0.81	1.20(0.63,2.26)	0.58	1.19(0.62,2.27)	0.60
Q3	0.91(0.59,1.43)	0.69	0.79(0.47,1.34)	0.38	1.07(0.51,2.25)	0.85	1.28(0.58,2.81)	0.54	1.26(0.56,2.85)	0.58

Q4	0.52(0.31,0.87)	0.014	0.75(0.40,1.39)	0.35	1.08(0.43,2.71)	0.88	1.35(0.49,3.69)	0.56	1.31(0.45,3.84)	0.63
P for trend	0.002		0.48		1		0.94		0.95	

Model 1:adjusted for age and gender;
Model 2:adjusted for multivariate variables: age, gender, race, waist circumference, education level, marital status, smoking, HDL-C, TC, eGFR, and comorbidities (hypertension, diabetes, stroke, and cardiovascular disease);
Model 3:adjusted for Model 2 and BMI;
Model 4:adjusted for Model 3 and MAMC;
Abbreviations: Q, quartiles; HR, hazard ratio; CI, confidence interval.

Supplementary Table 3 | Cox regression analysis between MUAC and all-cause, cardiovascular as well as cerebrovascular mortality

	Unadjusted<br>HR(95% CI),P-value	Model 1<br>HR(95% CI),P-value	Model 2<br>HR(95% CI),P-value	Model 3<br>HR(95% CI),P-value
**All-cause mortality**				
MUAC (per cm increment)	0.95(0.94,0.96) <0.001	0.96(0.95,0.97) <0.001	0.90(0.89,0.92) <0.001	0.89(0.87,0.91) <0.001
MUAC quartiles				
Q1	Reference	Reference	Reference	Reference
Q2	0.81(0.75,0.89) <0.001	0.74(0.68,0.81) <0.001	0.64(0.57,0.71) <0.001	0.66(0.59,0.74) <0.001
Q3	0.69(0.63,0.75) <0.001	0.66(0.61,0.73) <0.001	0.52(0.46,0.60) <0.001	0.56(0.48,0.64) <0.001
Q4	0.56(0.51,0.61) <0.001	0.64(0.58,0.71) <0.001	0.43(0.36,0.52) <0.001	0.48(0.40,0.59) <0.001
P for trend	<0.001	<0.001	<0.001	<0.001
**Cardiovascular mortality**				
MUAC (per cm increment)	0.95(0.92,0.99) 0.007	0.97(0.95,0.99) 0.001	0.89(0.85,0.92) <0.001	0.86(0.82,0.90) <0.001

MUAC quartiles				
Q1	Reference	Reference	Reference	Reference
Q2	0.81(0.66,0.99) 0.035	0.74(0.60,0.91) 0.004	0.58(0.45,0.75) <0.001	0.59(0.45,0.76) <0.001
Q3	0.58(0.47,0.73) <0.001	0.59(0.47,0.74) <0.001	0.39(0.28,0.53) <0.001	0.39(0.28,0.55) <0.001
Q4	0.59(0.47,0.73) <0.001	0.76(0.60,0.96) 0.023	0.36(0.24,0.53) <0.001	0.36(0.23,0.56) <0.001
P for trend	<0.001	<0.001	<0.001	<0.001
**Cerebrovascular mortality**				
MUAC (per cm increment)	0.95(0.92,0.99) 0.007	0.99(0.95,1.03) 0.60	1.00(0.92,1.08) 0.99	1.02(0.92,1.13) 0.79
MUAC quartiles				
Q1	Reference	Reference	Reference	Reference
Q2	1.11(0.74,1.67) 0.61	1.16(0.77,1.75) 0.48	0.99(0.55,1.77) 0.97	0.99(0.54,1.83) 0.97
Q3	0.86(0.56,1.32) 0.48	1.05(0.68,1.64) 0.82	1.14(0.59,2.23) 0.70	1.15(0.56,2.39) 0.71
Q4	0.51(0.31,0.86) 0.10	0.83(0.49,1.42) 0.50	0.82(0.33,2.03) 0.67	0.85(0.30,2.41) 0.77

P for trend	0.021	0.64	0.79	0.84

Model 1:adjusted for age and gender;
Model 2:adjusted for multivariate variables: age, gender, race, waist circumference, education level, marital status, smoking, HDL-C, TC, eGFR, and comorbidities (hypertension, diabetes, stroke, and cardiovascular disease);
Model 3:adjusted for Model 2 and BMI;
Abbreviations: Q, quartiles; HR, hazard ratio; CI, confidence interval.

1.2 Supplementary Figures

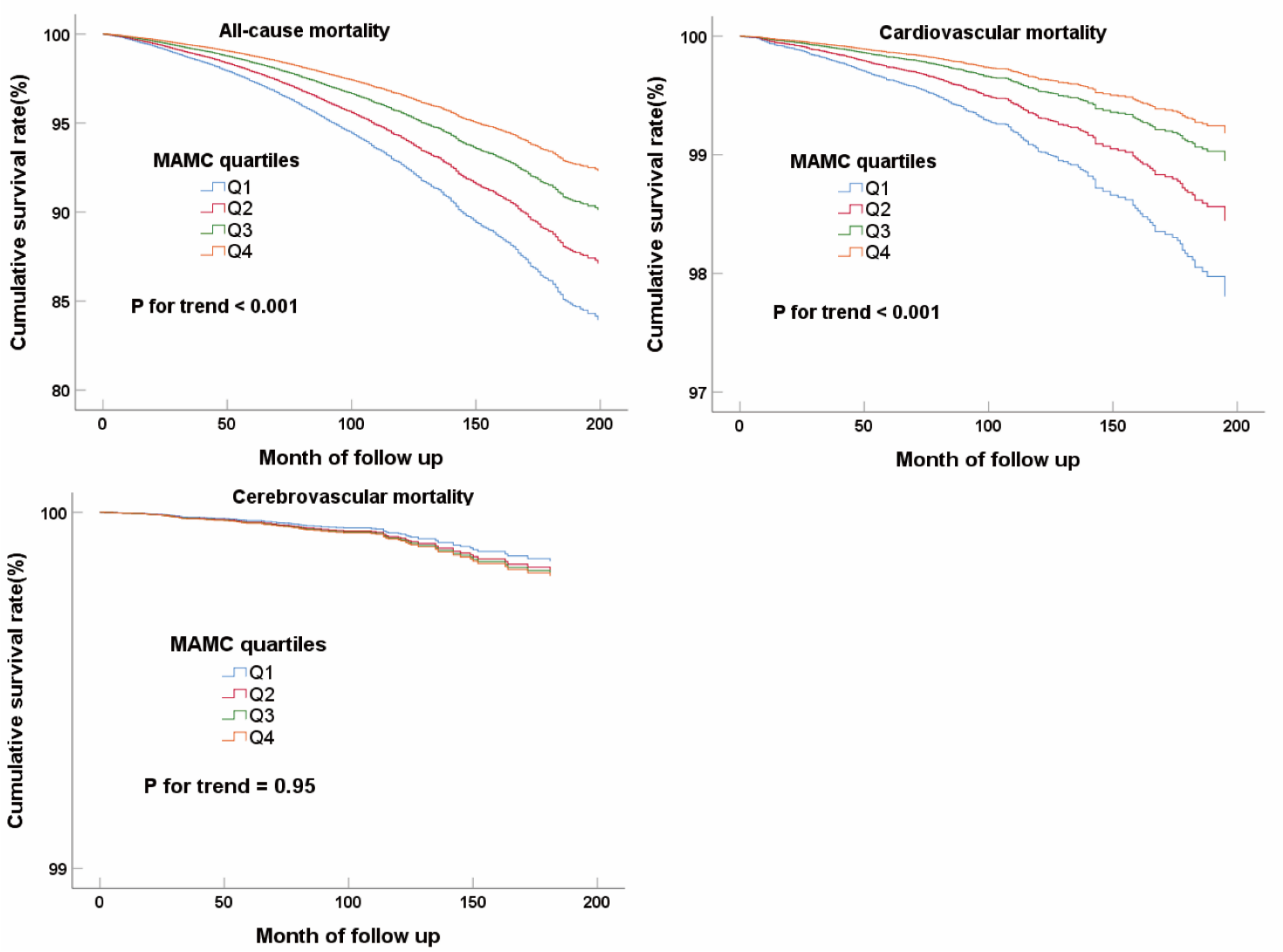

Supplementary FIGURE 1 | Cox regression curves for all-cause (A), cardiovascular (B) and cerebrovascular (C) mortality according to the quartiles of adult MAMC after adjusting for Model 4

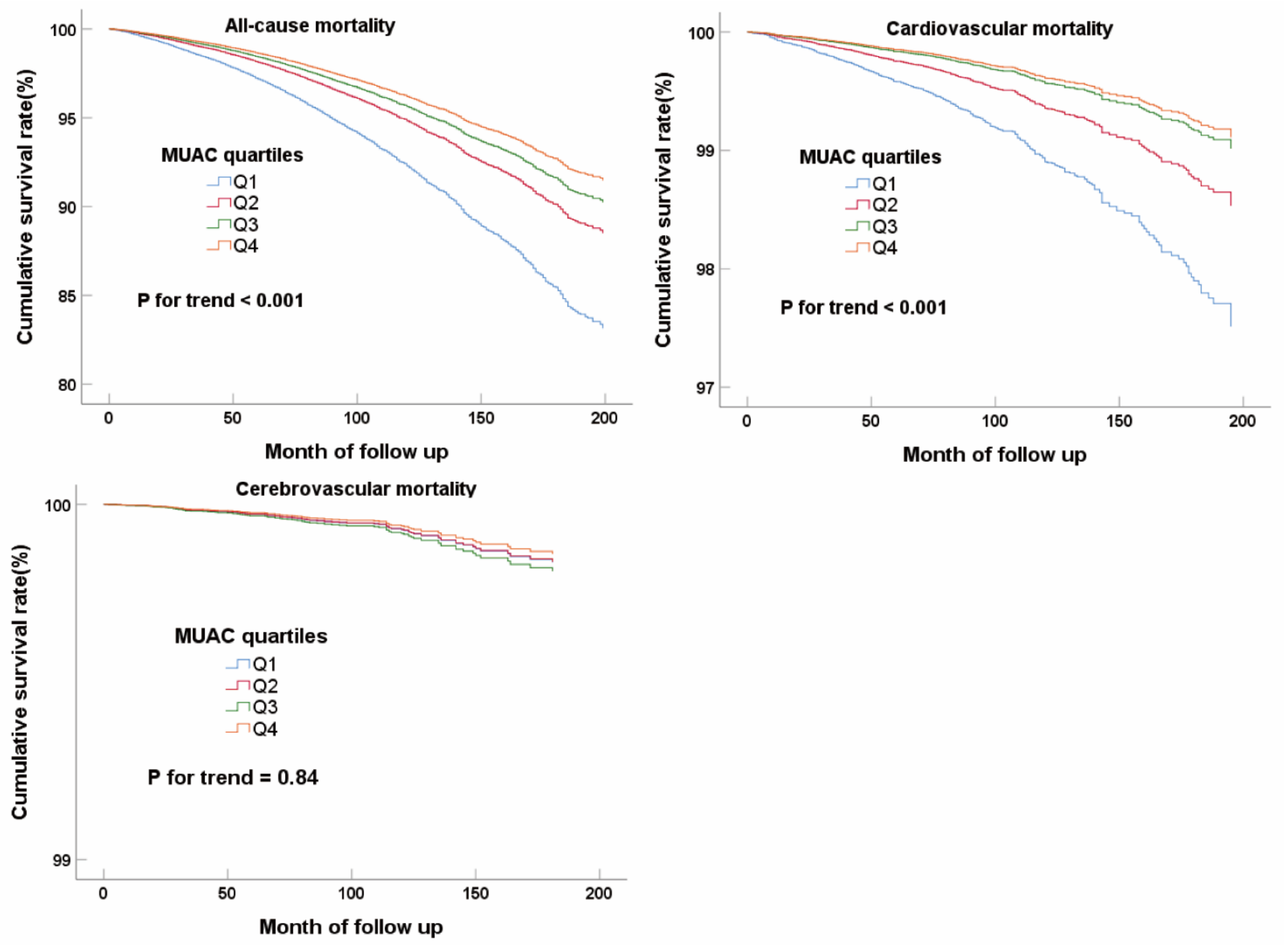

Supplementary FIGURE 2 | Cox regression curves for all-cause (A), cardiovascular (B) and cerebrovascular (C) mortality according to the quartiles of adult MUAC after adjusting for Model 4
